# Factors Associated With HIV Testing Among MSM in Guilin, China: Results From a Cross-Sectional Study

**DOI:** 10.3389/ijph.2022.1604612

**Published:** 2022-07-20

**Authors:** Jianfang Zhou, Lu Yang, Jingyi Ma, Shenyue Jiang, Yuelong Liu, Zhiming Sun

**Affiliations:** ^1^ Nanjing University of Posts and Telecommunications, Nanjing, China; ^2^ Guilin Center for Disease Control and Prevention, Guilin, China; ^3^ Jiangsu Health Development Research Center, Nanjing, China

**Keywords:** China, HIV/AIDS, MSM, HIV testing, men have sex with men

## Abstract

**Objectives:** The objective of this study is to explore factors affecting the HIV testing behaviors among men who have sex with men (MSM) in China.

**Methods:** A cross-sectional study was conducted in Guilin, China from April to June of 2021. Questionnaire data of 300 MSM were analyzed, and binary logistic regression models were used to examine the socio-demographic and sexual behavior characteristics associated with three HIV testing behaviors (self-testing, institutional testing, and regular testing).

**Results:** The results showed that half of the respondents had the habit of regular HIV testing. Only 30.0% of MSM chose to do HIV testing after high-risk sexual behavior, and self-perceived luck was the main reason for not having HIV testing. Moreover, the influencing factors of three HIV testing behaviors after high-risk sexual behavior differ. Interestingly, income was not related to any of the three HIV testing behaviors among those MSM who participated.

**Conclusion:** This research indicates insufficient health education on HIV testing behaviors among MSM in China. Health promotion practices targeting the MSM population to improve HIV-related knowledge, thus contributing to the HIV epidemic, are required.

## Introduction

According to the Global AIDS Update 2021, human immunodeficiency virus (HIV) remains a global health crisis. There were around 1.5 million people newly infected with HIV in 2020 worldwide [1]. Among the new infections outside sub-Saharan Africa, gay men and men who have sex with men (MSM) made up the highest proportion, accounting for 45% of the total [1]. Moreover, according to China CDC’s national data on HIV/AIDS, a rising trend of HIV prevalence has been identified among the Chinese MSM population, ranging from 6.9% in 2019 to 8.0% in 2020 [2, 3]. Ending the epidemics of AIDS is one of the health targets of the Sustainable Development Goals (SDGs) [4].

Since 2003, China has introduced the “Four Frees and One Care” policy to provide free HIV Voluntary Counseling and Testing (VCT) services nationwide. In 2013, the Joint United Nations Program on HIV/AIDS (UNAIDS) 90-90-90 goals proposed that 90% of all people living with HIV should know their HIV status, 90% of those diagnosed should receive antiretroviral therapy (ART), and 90% of those should have durable viral suppression, in order to help end the AIDS epidemic [5]. Literature shows that China has progressed towards UNAIDS 90-90-90 targets among MSM [2]. However, socio-structural barriers such as HIV-related stigma, concerns regarding confidentiality, etc., have led to low uptake of HIV testing among MSM [6]. Therefore, China’s disease control system has widely publicized information on HIV testing, including promoting these tests in pharmacies and social media platforms, and distributing self-testing kits free of charge in the CDC. As a result, those efforts enhance the accessibility and convenience of HIV testing. The latest data shows that in 2019, 56.40% of MSM had been tested and knew their infection status [7]. Data from MSM surveys conducted in the past 2 years showed that the testing rate ranged from 48.6%–83.8% [8–12] in some Chinese provinces.

Fully understanding the factors affecting the HIV testing uptake among the MSM population and improving the HIV testing rate to reduce HIV incidence remain persistent challenges in China. Previous studies found that marital status, sexual behavior, HIV-related knowledge, HIV risk perception, and social stigma were the factors that attributed to the intention to get an HIV test among the MSM population [11, 13–17]. However, few studies focus on comprehensive HIV testing behaviors and their relationship with psychosocial characteristics among MSM [18–20]. As understanding the influencing factors associated with HIV testing behaviors among MSM is important to fully strengthen the accessibility and quality of HIV prevention programs, this study explores the impact of socio-demographic and psychosocial factors associated with HIV testing behaviors (institutional HIV testing after high-risk sexual behavior, self-testing after high-risk sexual behavior, and regular self-testing) among MSM, and investigates the reasons behind the decision of whether or not to undergo HIV testing among those MSM.

## Methods

### Study Design

A pilot questionnaire was tested on 20 MSM respondents in November 2020 to ensure the final version of the questionnaire was appropriate. From April to June 2021, the formal cross-sectional survey study was conducted in Guilin, China. Paper questionnaires were distributed and collected by ten trained investigators who all belonged to the local MSM peer group. After the investigator explained to the participants the purpose of the research, result utilization, privacy, and assured confidentiality of information were strictly guaranteed by all data collectors and investigators, and the participants completed the paper questionnaires anonymously. In the end, the participants returned with the sealed envelopes to the investigators.

### Setting

All the participants were recruited from Guilin City, Guangxi Province, China. Guangxi is one of the five provinces in China with the highest prevalence of HIV infection. By the end of 2020, more than 14,000 people with HIV had been reported and MSM have become the fastest-growing population at risk for the HIV epidemic in Guilin [21].

### Sampling

Participants were recruited using the convenient sampling method. Eligibility criteria included [1] being aged 18 years or older [2]; having sex with men in the last 12 months [3]; consenting to participate in the research [4]; visiting the clinic for VCT in Guilin CDC.

### Measure of Variables

There were three dependent variables: whether to go to the institution for HIV testing after high-risk sexual behavior, whether to perform an HIV self-testing after high-risk sexual behavior, and whether to take HIV tests regularly. All of them were measured as binary outcomes (yes or no).

The study investigated the possible associated factors found in previous studies, which include: demographic characteristics (including age, education level, whether they have steady partners, and income), sexual characteristics (including sexual identity, sexual roles, and the number of sexual partners in the past year), social stigma (whether they were afraid of others finding out they are MSM), and HIV infection status, HIV testing knowledge scores (six knowledge items, one point is given for every correct answer, a total score ranging from 0 to 6), and risk perception of HIV infection (very high risk, relatively high risk, relatively low risk, almost no risk, and no risk). Moreover, reasons for recent HIV testing and reasons for not having HIV testing after high-risk sexual behavior were investigated among participants.

### Data Management and Statistical Analysis

Multivariate logistic regression models were used to analyze all the factors of relevance influencing HIV testing behaviors, including demographic characteristics, social stigma, HIV infection status, HIV testing knowledge, and risk perception of HIV infection. 95% confidence level for the adjusted odds ratio (aOR 95% CI) was used to assess the interaction between possible influencing factors and dependent variables after controlling for confounding factors. The Hosmer–Lemeshow test was used to check for collinearity (*p* > 0.05). Factors with a *p*-value of less than 0.05 were identified as independently associated with HIV testing behavior. All statistical analyses were processed with SPSS.25.0.

## Results

### Characteristics of Respondents

Table 1 shows the demographic characteristics of participants. The mean age of those 300 MSM was 36. Almost half of those who participated in this study had University qualifications or higher (44.30%). More than half of those MSM had an income of more than 3,000 CNY per month. Regarding sexual characteristics, there were 144 (48.00%) MSM who chose homosexuality and 166 (55.30%) had a versatile sexual role. Moreover, there were 202 (67.30%) MSM who reported having social stigma due to their sexual orientation. For HIV-related concerns, 159 (53.00%) believed their risk of HIV infection was “very low,” and fifteen respondents (5.00%) were worried that others would know their HIV status. Furthermore, the average score of HIV testing knowledge was 3.8 among those 300 MSM who participated.

**TABLE 1:** Socio-demographic characteristics of men who have sex with men; China (2021).

Characteristics	Mean (SD) or n (%)	Characteristics	Mean (SD) or n (%)
Age	36.21 (13.45)	Sexual identity	
		homosexuality	144 (48.00)
		Bisexuality	130 (43.30)
		don’t know	28 (8.70)
Education level		Sexual role	
Junior high school or lower	60 (20.00)	Exclusively receptive (ER)	51 (17.00)
Senior high school	107 (35.70)	Exclusively insertive (EI)	83 (27.70)
University or higher	133 (44.30)	Role versatile (RV)	166 (55.30)
Income (¥)		HIV infection risk perception	
Less than 1,500	63 (21.00)	Very high risk	16 (5.30)
1,500-2,999	91 (27.00)	Relative high risk	40 (13.30)
3,000-5,999	118 (39.30)	Very low risk	159 (53.00)
6,000 and more	38 (12.70)	Relative low risk	54 (18.00)
		No risk	31 (10.30)
Number of sexual partners in the past year	4.31 (9.51)	HIV testing knowledge score	3.78 (1.43)
Worried that others will know HIV status		Social stigma	
Yes	15 (5.00)	Yes	202 (67.30)
No	285 (95.00)	No	98 (32.70)

### HIV Testing Behavior and Reasons for HIV Testing

Of the 300 respondents, 15 had undergone HIV testing for the first time. The reasons for the latest HIV testing and reasons for not having HIV testing after high-risk sexual behaviors among MSM (*n* = 285) are shown in Table 2. More than half of the total participants (*n* = 162, 54.00%) reported regular HIV testing. Receiving relevant promotional information (48.80%), engaging in high-risk sexual behaviors (24.60%), and being sick or feeling unwell (23.20%) were the top three reasons for the latest HIV testing.

**TABLE 2:** Reasons for the latest HIV testing and not having HIV testing among men who have sex with men after high-risk sexual behaviors (multiple-choice question); China (2021).

Reasons latest VCT use (*n* = 285)		Reasons for not having HIV tests after high-risk sexual behaviors (*n* = 210)	
Reason	n (%)	Reason	n (%)
Sick or feeling unwell	66 (23.20)	Self-perceived luck	163 (77.60)
Had a high-risk sexual behavior	70 (24.60)	Poor attitude of healthcare staff	22 (10.50)
New infections around	54 (18.90)	Afraid of others knowing the test result	15 (7.10)
Received relevant HIV testing promotional information	139 (48.80)	Not necessary for knowing the result	50 (23.80)
Received advice from sexual partner	28 (9.80)	No time to test	73 (34.80)
Other reasons	0 (0.00)	Don’t know where to get the test	28 (13.30)
		Worried about the cost of the test	44 (21.00)

When asking what they would do if they had engaged in any high-risk sexual behaviors (multiple-choice question), there were 273 participants (91.00%) who responded that they would take an institutional test, 135 (46.00%) may do self-testing, and only 90 (30.00%) chose that they would definitely do an HIV test. Moreover, among those who reported not having HIV testing after high-risk sexual behaviors (*n* = 210, 70.00%), self-perceived luck (77.60%), lack of time (34.80%), and no perceived necessity of knowing the HIV test result (23.80%) were the main reasons for not having HIV testing.

### Factors Associated With MSM HIV Testing Behaviors

Table 3 shows the results of the multivariate logistic regression models. All three models passed the collinearity test (*p* > 0.05). The sexual role, social stigma, and HIV testing knowledge score were associated with having an institutional HIV test after high-risk sexual behavior. Specifically, those with a sexual role of versatile (aOR = 0.29, 95% CI 0.11–0.75), those who were not afraid of others finding out (aOR = 3.02, 95% CI 1.10–6.92), and those with higher HIV testing knowledge scores (aOR = 1.48, 95% CI 1.03–2.13) were more likely to go to the CDC or hospital for an HIV test after high-risk sexual behaviors. Moreover, the age and number of sexual partners in the last 12 months were associated with undergoing HIV self-testing after high-risk behavior. Those who were younger (aOR = 0.98, 95% CI 0.95–1.00) and had more sexual partners (aOR = 1.17, 95% CI 1.00–1.37) in the last 12 months were more likely to undergo HIV self-testing after engaging in high-risk behavior. Furthermore, MSM undergoing regular HIV testing were associated with having university or higher-level education qualifications (aOR = 2.26, 95% CI 1.01–5.03) and self-perceived higher risk of HIV infection (aOR = 0.35, 95% CI 0.13–0.98).

**TABLE 3:** Factors associated with three HIV testing behaviors: Results of multivariate logistic regression analyses; China (2021).

Variable (control group)	Model 1: Going to CDC or hospital for an HIV test after engaging in high-risk behavior			Model 2: HIV self-testing after engaging in high-risk behavior			Model 3: Habit of regular HIV testing		
	aOR	aOR 95% CI		aOR	aOR 95%CI		aOR	aOR 95% CI	
		Lower	Upper		Lower	Upper		Lower	Upper
Age (years)	0.99	0.95	1.03	**0.98**	**0.95**	**1.00**	1.01	0.99	1.03
Education level (university or higher)									
Junior middle school or lower	0.35	0.09	1.39	0.55	0.25	1.19	**2.26**	**1.01**	**5.03**
Senior middle school	0.82	0.25	2.73	0.71	0.40	1.28	0.63	0.35	1.14
Income (≥¥6,000)									
<¥1,500	0.20	0.02	2.08	0.82	0.32	2.11	0.61	0.24	1.57
¥1,500-2,999	0.41	0.04	3.97	0.70	0.29	1.68	0.98	0.41	2.37
¥3,000-5,999	0.49	0.05	4.47	1.48	0.68	3.24	1.21	0.55	2.66
Sexual identity (homosexuality)									
Don’t know	2.13	0.40	11.34	0.45	0.17	1.16	**0.35**	**0.13**	**0.98**
Bisexuality	1.96	0.67	5.75	0.82	0.46	1.47	1.36	0.77	2.41
Sexual role (RV)									
ER	2.00	0.38	10.60	1.99	0.95	4.18	0.70	0.33	1.46
EI	**0.29**	**0.11**	**0.75**	1.42	0.78	2.57	1.01	0.56	1.84
Number of sexual partners in the past year	1.21	0.89	1.66	**1.17**	**1.00**	**1.37**	0.89	0.76	1.04
MSM stigma (yes)	**3.02**	**1.10**	**6.92**	1.06	0.60	1.87	0.62	0.35	1.09
HIV infection risk perception (no risk)									
Very high risk	0.61	0.08	4.61	0.39	0.10	1.53	0.38	0.09	1.53
Relative high risk	0.29	0.06	1.45	0.39	0.13	1.14	0.51	0.17	1.56
Relative low risk	1.13	0.26	4.92	0.84	0.35	2.03	0.48	0.18	1.24
Very low risk	2.03	0.33	12.53	0.62	0.23	1.67	**0.33**	**0.12**	**0.96**
HIV testing knowledge score	**1.48**	**1.03**	**2.13**	1.17	0.97	1.41	1.01	0.84	1.22
Worried that others will know the result of the infection (no)	0.58	0.06	5.96	1.36	0.43	4.32	2.13	0.66	6.84
Model	Hosmer-Lemeshow test *p* = 0.43; R^2^ = 0.27			Hosmer-Lemeshow test *p* = 0.45; R^2^ = 0.19			Hosmer-Lemeshow test *p* = 0.73; R^2^ = 0.19		

Note: aOR, adjusted odds ratio; Each model included all variables of relevance.

## Discussion

This is the first research to analyze three HIV testing behaviors together and their associations among MSM in Guilin, China. The results from this research showed a relatively higher rate of regular HIV testing, institutional HIV testing, and self-testing after high-risk sexual behavior among MSM compared to previous studies [22–24], which may be related to the widely available and readily accessible human immunodeficiency virus self-testing (HIVST) in China [25]. The study showed that the proportion of MSM who went to institutions for HIV testing after high-risk sexual behavior was greater than that of MSM who selected self-testing. This is consistent with findings from previous studies [23, 26]. However, this study revealed that the proportion of MSM who chose definitely to undergo the HIV test after each high-risk sexual behavior was only 30.0%, which indicates that there is still a big gap to achieve the 90-90-90 HIV Target in confirming HIV status. Furthermore, this research showed that most participants did not have HIV testing due to their self-perceived luck, indicating that health promotion and education programs implemented with self-testing in the high-risk populations are needed. An Internet-based HIV self-testing program has proven feasible and acceptable in increasing HIV testing uptake among MSM in Brazil [27], presenting possibilities of E-testing for MSM in China. For example, a web-based narrative communication intervention to promote HIV-preventive behaviors among MSM can be incorporated, which was indicated to be easily replicable [28].

The results from this study showed that HIV testing knowledge score was associated with having institutional HIV testing after high-risk sexual behavior among MSM, which is in accordance with a US study comprising 374 MSM from Atlanta, indicating that greater HIV knowledge was associated with greater likelihood and frequency of HIV testing [29]. As knowledge is critical in making health decisions, and the knowledge of HIV has been studied and found to be associated with reduced high-risk sexual behaviors among MSM [30], HIV/AIDS interventions including HIV knowledge education are required. However, most studies on HIV/AIDS interventions with a focus on the influence of HIV testing services among the MSM population were conducted without integrating the role of HIV testing knowledge education and health consultation [31–33]. Even the UNAIDS 90-90-90 HIV Target has overlooked the role of HIV testing knowledge. Due to a strong link between HIV-related knowledge and HIV testing, it is suggested that various forms of HIV testing education are still needed. For example, pop-up advertising regarding HIV testing knowledge on social media can be promoted based on the fact that a majority of MSM used social networking applications to find sexual partners [34].

It is interesting to notice from this research that income was not one of the confounding factors influencing HIV testing behaviors among MSM, which is inconsistent with previous research [35–37]. The reason may be related to the promotion of HIV VCT services and the affordability of self-testing reagents in China (approximately 5 USD per use in Guilin). As such, it can be challenging to increase the HIV testing rate only by providing free or low-cost HIV testing services among Chinese MSM. Moreover, a systematic review indicated that free or low-cost HIV self-testing services could be much more effective in enlarging HIV testing in resource-limited countries than in high-income countries [38]. However, there is insufficient evidence to draw a conclusion regarding the association between income and HIV testing behaviors among MSM. More data from high-quality research is needed to evaluate the effects of income and HIV testing cost in HIV interventions among MSM.

This study revealed that the influencing factors of HIV typical testing behavior and self-testing after high-risk sexual behavior differ. The decision to undergo an institutional test after high-risk behavior is related to the sexual role, social stigma, and HIV knowledge. The decision to undergo HIV self-testing after high-risk behavior was related to age and the number of sexual partners, while regular testing behavior is related to education, sexual identity, and risk perception. Awareness of HIV status is an essential step in prevention, care, and treatment services. This research suggests that when promoting HIV testing among the MSM population, different strategies are needed to promote specific HIV testing behaviors. For example, publicizing HIV self-testing for MSM who are relatively older. When it comes to increasing regular testing, reinforcing the importance of focusing prevention efforts on MSM with a higher level of education and lower level of risk perception is suggested. Moreover, implementing institutional policies that address social stigma would be helpful for improving institutional HIV testing and would emphasize proper self-evaluation of infection risk to help improve the level of regular testing. In the future, more context-based health education campaigns and comprehensive health promotion strategies, such as a syndetic approach [39] to HIV prevention, are required.

This study has several limitations. First, study participants were outpatients recruited from CDC, so the institutional HIV testing rate after high-risk sexual behavior and the regular testing rate may be higher than the HIV testing rate among MSM recruited from other sources. Second, is the research utilized a cross-sectional design that may not be sufficient to understand HIV testing trends. Nevertheless, this research is the first to reveal three HIV testing behaviors and their influencing factors among MSM in China, providing insights on how to address the MSM population’s concerns regarding HIV testing interventions and health education.

### Conclusion

Although VCT and self-testing reagents are widely promoted in China, the HIV testing rate among MSM is lower than the UNAIDS 90-90-90 goal. Self-perceived luck is the main reason MSM did not undergo HIV testing after high-risk sexual behavior. Age, education, the number of sexual partners, sexual identity, sexual role, risk perception of HIV infection, and HIV testing knowledge were factors related to HIV testing. The results presented in this research point to the need for HIV prevention and education efforts designed for the high-risk MSM population.
